# Extravasation Mucocele Arising from a Lingual Thyroglossal Duct Remnant

**DOI:** 10.1155/2015/326251

**Published:** 2015-03-12

**Authors:** Mitsuhiko Nakahira, Hiroaki Nakatani

**Affiliations:** ^1^Department of Head and Neck Surgery, Saitama Medical University International Medical Center, 1397-1 Yamane, Hidaka, Saitama 350-1298, Japan; ^2^Department of Otolaryngology, National Hospital Organization Fukuyama Medical Center, 4-14-17 Okinogamicho, Fukuyama, Hiroshima 720-0825, Japan

## Abstract

Although a thyroglossal duct cyst is a congenital anomaly, it can also appear in adults. Despite the presence of embryological remnants, it is still unclear why the cyst should suddenly develop later in life. We report a case of a 46-year-old male with an extravasation mucocele arising from a long-standing lingual thyroglossal duct remnant. MRI demonstrated a lingual cystic lesion near the hyoid bone associated with a suprahyoid tract-like structure masquerading as a thyroglossal duct cyst. However, histopathological examination demonstrated a mucocele secondary to a rupture of a thyroglossal duct remnant with numerous intramural heterotopic salivary glands. We propose a new mechanism of an acquired cystic formation of this congenital disease that excessive production of mucus from heterotopic salivary glands and a physical trauma such as swallowing may lead to extravasation of mucus from the thyroglossal duct.

## 1. Introduction

A cyst of the thyroglossal duct is the most common congenital neck mass [1]. Although a thyroglossal duct cyst is a congenital anomaly and most commonly presents in a pediatric age group, it can also appear in adults [2]. Recent studies have shown that thyroglossal duct remnants are far more common than thyroglossal duct cysts [3]. The key factors involved in the pathogenesis of an acquired cystic formation from a thyroglossal duct remnant, however, are largely unknown [1, 4]. An extravasation mucocele is an acquired cystic abnormality in the accessory salivary glands [5]. Both extravasation mucocele and thyroglossal duct cysts are considered different pathological entities in the head and neck [6]. In the present report, we describe a rare case of an extravasation mucocele arising from a long-standing lingual thyroglossal duct remnant.

## 2. Case Report

**Figure 1:**
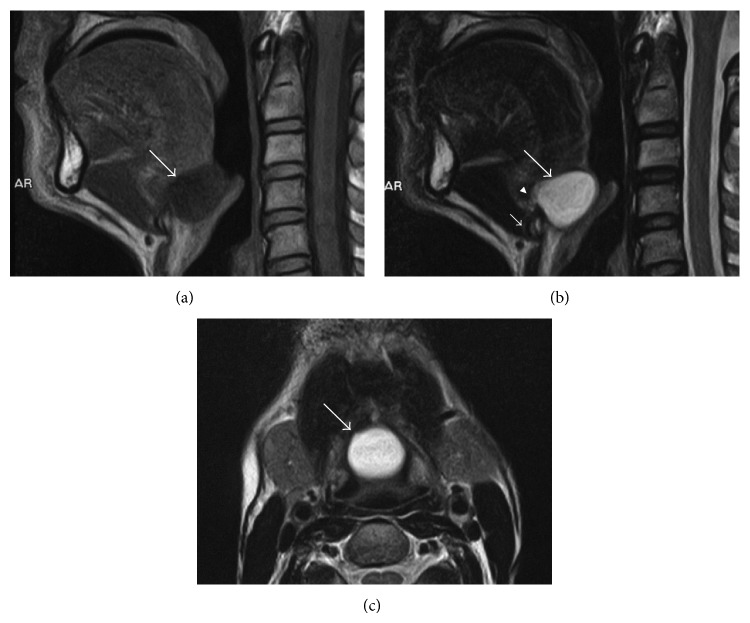
(a) Sagittal T1-weighted (TR/TE, 585/10), (b) sagittal T2-weighted (TR/TE, 4000/100), and (c) axial T2-weighted (TR/TE, 4000/100) MR images of the neck. The round mass (large arrow) has a homogenous low signal intensity in (a) and a high signal intensity in (b) and (c), suggesting a cystic lesion. In (b), there is a black and white striped tract (arrowhead) from the hyoid bone (small arrow) to the mass (large arrow), suggesting the thyroglossal duct.

**Figure 2:**
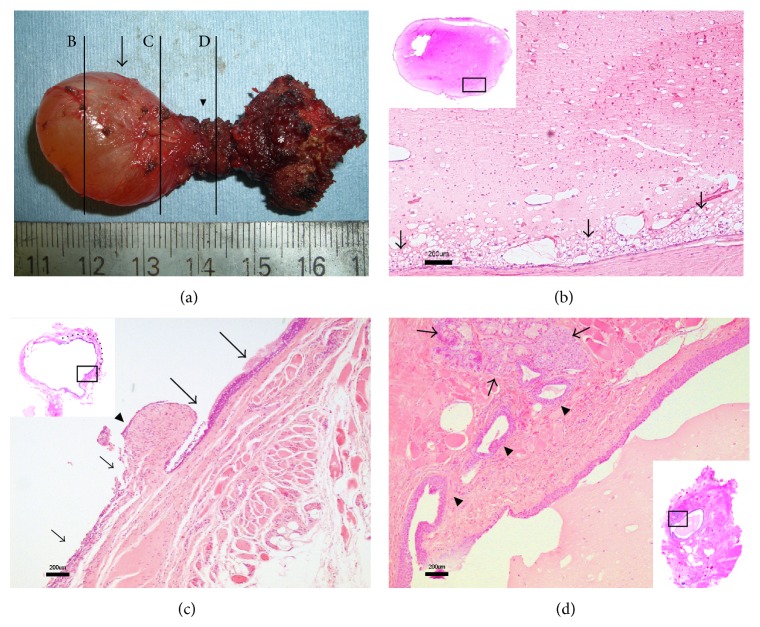
The thyroglossal duct and cyst. (a) The extirpated specimen consists of a 20 × 20 × 15 mm cyst (arrow), the bridging tract (arrowhead), and the middle third of the hyoid bone (asterisk). The perpendicular lines indicate the following three sections (B, C, and D). (b) Extravasation mucocele. Microscopic examination of the specimens through line B shows a nonepithelial lining cyst (hematoxylin-eosin stain; bar = 200 *μ*m). Note that there are numerous foamy histiocytes (arrows) adjacent to the nonepithelial lining wall of the cyst. (c) Associated ruptured cyst. Microscopic examination of the specimens through line C shows an associated ruptured cyst (hematoxylin-eosin stain; bar = 200 *μ*m). This section is composed of two different parts: the ciliated respiratory epithelium (large arrows) and the no epithelial lining (small arrows). Note the puckered distinct border between these parts (arrowhead), which might have resulted from the rupture of the cyst. The dotted line indicates the presence of the epithelial lining. (d) Thyroglossal duct. Microscopic examination of the specimens through line D shows the thyroglossal duct with transitional epithelium lining and the presence of heterotopic salivary glands (arrows) and ducts (arrowheads) draining into the thyroglossal duct (hematoxylin-eosin stain; bar = 200 *μ*m).

A 46-year-old male with a two-year history of globus sensation was referred to our hospital because of a tumor at the base of his tongue. He did not complain of sore throat or dysphagia. His voice sounded normal and no tumor was found in the neck. His symptoms gradually worsened, especially in the supine position. Flexible fiber-optic examination revealed a smooth round mass in the midline of the base of the tongue. MRI of the neck revealed a well-circumscribed cystic lesion coupled with an ascending tract from the hyoid bone towards the base of the tongue (Figures 1(a)–1(c)). The thyroid gland was identified in the normal position in the neck. The patient underwent a Sistrunk procedure for a presumed thyroglossal duct cyst. The complete specimen containing a cyst, the bridging tract, and the middle third of the hyoid bone were extirpated without any entry into the pharynx (Figure 2(a)). Histopathological examination demonstrated that the lesion was composed of a non-epithelial-lined cyst, an associated ruptured cyst, and an epithelial-lined thyroglossal duct remnant containing numerous heterotopic salivary glands in the duct wall (Figures 2(b)–2(d)). Because the sequential lesion had the ruptured cyst between the cyst and the thyroglossal duct remnant, the cyst was considered as an extravasation mucocele from the thyroglossal duct remnant. The patients' postoperative course was uneventful. He commenced oral intake on the first postoperative day. Six-month follow-up revealed no evidence of recurrence and no new relevant symptoms.

## 3. Discussion

This patient explains the phenomenon of delayed presentation of a cyst from an embryological remnant after having been dormant in the neck for years. The thyroglossal duct is an embryological remnant of the pathway taken by the thyroid anlage as it descends from the region of the foramen cecum in the back of the tongue to its final position low in the neck. This duct normally disappears by fifth to tenth week of life [1]. If a portion of the duct persists, a cyst may occur, presumably as a result of the secretion of colloid-like material from its epithelial lining. Despite the presence of embryological remnants, it remains unclear why a cyst should suddenly develop later in life [1, 4]. The cyst is generally thought to arise the thyroglossal duct remnant itself; a proliferation of these remnants may occur due to various etiologic factors, including inflammation, the most hypothesized initiating stimulus, and infections such as colds, which may stimulate the epithelial remnants of the thyroglossal tract to undergo cystic changes. It is also possible that a blocked thyroglossal duct could be responsible for the cystic expansion. Our case suggests another potential pathophysiological explanation.

A cyst may develop anywhere along the course of the thyroglossal duct; however, only 2% are found at the base of the tongue [1]. There are a number of potential diagnoses to be considered for masses that appear at the base of the tongue, including lingual thyroid glands, salivary gland tumors, vallecular cysts, lingual dermoid cysts, and mucous cysts. In the present report, MR images indicated a cyst arising within a lingual thyroglossal duct remnant. Additionally, although the suprahyoid remnant of the thyroglossal duct is infrequently seen on MR images [7], the suprahyoid thyroglossal duct was clearly identified as an ascending tract-like structure from the hyoid bone in our case. Despite MR images showing a cystic lesion arising from a thyroglossal duct, it is impossible to distinguish a true cyst from a pseudocyst. The definitive diagnosis should be made by histopathological examination, which can reveal whether a cystic lesion has an epithelial lining along its lumen. Thyroglossal duct cysts occasionally lose their epithelial linings due to infection or other causes [1, 4]. Although the cyst in our patient had no-epithelial-lining, the lesion had a ruptured cyst between a mucocele and a thyroglossal duct. We consider this decisive evidence of extravasation of mucus from the thyroglossal duct.

**Figure 3:**
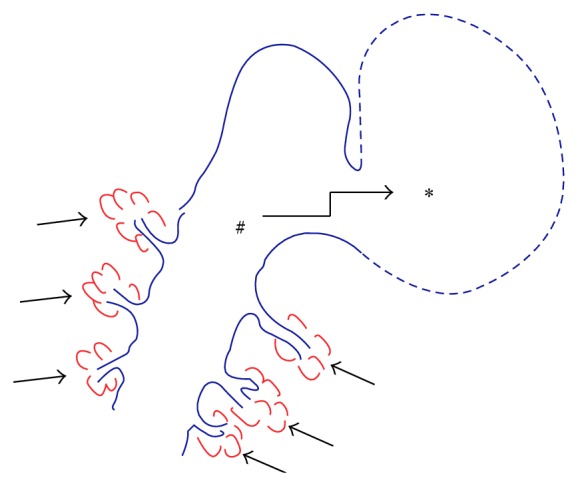
A possible pathophysiological mechanism of this condition. Excessive production of mucus into the thyroglossal duct (number sign) from the intramural heterotopic salivary glands (arrows) and a physical trauma such as swallowing may lead to extravasation of mucus (asterisk) secondary to a rupture of the thyroglossal duct.

Salivary gland tissue is reported within the thyroglossal duct remnant in 1.6–60% [4, 8] of patients and is seen most frequently identified in the lingual area. The accessory salivary glands and ducts draining into the suprahyoid thyroglossal duct are considered a possible cause of thyroglossal duct cyst recurrence after surgery [9]. Moreover, it has been shown in human cadavers that numerous salivary glands around the lingual thyroglossal duct remnant have a potential role as a fourth major salivary gland complex [10]. An extravasation of mucus from accessory salivary glands results in a mucocele, which is often seen as an acquired cystic abnormality in the oral cavity [5]. Thus, speculatively, excessive production of mucus into the thyroglossal duct from the intramural heterotopic salivary glands and a physical trauma such as swallowing may lead to extravasation of mucus from the thyroglossal duct (Figure 3).

In conclusion, we report a case of a 46-year-old male with an extravasation mucocele arising from a long-standing lingual thyroglossal duct remnant, which was masquerading as a thyroglossal duct cyst on MRI. This finding suggests a possible mechanism of late-onset cystic development from the persistent thyroglossal duct remnant in the tongue.
